# Neuroprotective levels of IGF-1 exacerbate epileptogenesis after brain injury

**DOI:** 10.1038/srep32095

**Published:** 2016-08-26

**Authors:** Yu Song, Corrin Pimentel, Katherine Walters, Lauren Boller, Shabnam Ghiasvand, Jing Liu, Kevin J. Staley, Yevgeny Berdichevsky

**Affiliations:** 1 Bioengineering Program, Lehigh University, Bethlehem, PA 18015, USA; 2 Integrated Degree in Engineering, Arts, and Sciences (IDEAS) Program, Lehigh University, PA 18015, USA; 3 Department of Electrical and Computer Engineering, Lehigh University, Bethlehem, PA 18015, USA; 4 Department of Neurology, Massachusetts General Hospital, Boston, MA 02129, USA; 5 Harvard Medical School, Boston, MA 02129, USA.

## Abstract

Exogenous Insulin-Like Growth Factor-1 (IGF-1) is neuroprotective in animal models of brain injury, and has been considered as a potential therapeutic. Akt-mTOR and MAPK are downstream targets of IGF-1 signaling that are activated after brain injury. However, both brain injury and mTOR are linked to epilepsy, raising the possibility that IGF-1 may be epileptogenic. Here, we considered the role of IGF-1 in development of epilepsy after brain injury, using the organotypic hippocampal culture model of post-traumatic epileptogenesis. We found that IGF-1 was neuroprotective within a few days of injury but that long-term IGF-1 treatment was pro-epileptic. Pro-epileptic effects of IGF-1 were mediated by Akt-mTOR signaling. We also found that IGF-1 – mediated increase in epileptic activity led to neurotoxicity. The dualistic nature of effects of IGF-1 treatment demonstrates that anabolic enhancement through IGF-1 activation of mTOR cascade can be beneficial or harmful depending on the stage of the disease. Our findings suggest that epilepsy risk may need to be considered in the design of neuroprotective treatments for brain injury.

Insulin-Like Growth Factor-1 (IGF-1) signaling is involved in neural differentiation, survival, and response to brain injury^1^,^2^. IGF-1 receptor (IGF-1R) mRNA is abundant during development and remains highly expressed in mature brain^3^. Expression of IGF-1 decreases significantly after maturation in most neurons^4^.

Brain injury leads to significant alterations in expression of IGF-1 in reactive astrocytes^5^,^6^, microglia^7^,^8^, and neurons^9^. There is also a significant elevation in IGF-1R phosphorylation after experimental traumatic brain injury (TBI)^10^. Exogenously applied IGF-1 was found to be neuroprotective in animal models of hypoxic-ischemic and traumatic brain injuries^11^,^12^. These findings led to the suggestion that IGF-1 or its agonists may be used as therapeutics to improve outcomes following brain injury^13^,^14^,^15^,^16^,^17^.

Activation of IGF-1 receptor includes the phosphoinositide 3-kinase (PI3K)-Akt and RAS-mitogen-activated protein kinase (MAPK) pathways, which lead to modulation of gene transcription, protein synthesis, apoptosis, and other key cellular processes^1^,^2^. Increased phosphorylation of Akt and MAPK was found in animal models of brain injury, suggesting that these kinases mediate downstream effects of elevated IGF-1^18^.

One of the effectors of Akt signaling is the mTOR cascade^20^, and traumatic brain injury was found to cause changes in the mTOR pathway, including an increase in phosphorylation of ribosomal S6 protein^19^,^21^,^22^. The mTOR signaling cascade has also been implicated in epilepsy^23^,^24^,^25^. Mutations in genes that regulate mTOR are associated with epilepsy-linked focal malformations of cortical development, including tuberous sclerosis complex^26^,^27^,^28^. Seizures generated in an animal model of tuberous sclerosis complex, a genetic disorder in which mTOR is constitutively active, were suppressed by mTOR inhibitor rapamycin^29^,^30^. mTOR inhibition was also effective in reducing spontaneous seizures in some, but not all models of acquired epilepsy (reviewed by Goldberg and Coulter^24^ as well as Ostendorf and Wong^25^). Importantly, in an animal model of TBI and posttraumatic epileptogenesis, the mTOR inhibitor rapamycin decreased the seizure frequency and rate of development of posttraumatic epilepsy^22^. In the organotypic culture model of post-traumatic epileptogenesis, mTOR activation was mediated by PI3K-Akt pathway suggesting that growth factor signaling may be involved^31^.

Brain insults, including trauma, stroke, and infection, are the most prevalent known causes of acquired epilepsy^32^. Curiously, there is some evidence that neuroprotection can be associated with increased seizure activity. For example, successful thrombolytic treatment of stroke is a robust risk factor for epileptic activity^33^. Since brain injury is associated with changes in IGF-1 signaling, and one of the downstream effectors of IGF-1 signaling, mTOR pathway, is involved in epileptogenesis, it is possible that neuroprotective levels of IGF-1 may play a role in the development of epilepsy. Alterations in IGF-1 signaling that follow brain injury are transient whereas epileptogenesis occurs over a longer time scale; however, inhibition of transient mTOR activation after experimental TBI was found to be antiepileptogenic^22^. Therefore, injury-induced elevation in IGF-1 may play a role in epileptogenesis by contributing to mTOR activation. Chronic application of IGF-1 or its analogues as a treatment for brain injury may also inadvertently contribute to development of epilepsy through a similar mechanism. To examine the potential role of IGF-1 in epilepsy, we used organotypic hippocampal culture model of epileptogenesis^34^,^35^. In this *in vitro* model, the critical features of clinical epileptogenesis are captured on a compressed scale: latent period after injury characterized by axon sprouting, followed by gradual onset of population spiking activity and then spontaneous electrographic seizures, seizure clustering and status epilepticus that leads to activity-dependent neuron death^36^ (Fig. 1).

## Results

### IGF-1 was neuroprotective immediately after injury

We used confocal microscopy to evaluate and compare numbers of neurons in organotypic hippocampal cultures when IGF-1 was included in medium immediately after trauma (slicing) on days *in vitro* (DIV) 0–3. We found that cultures maintained in the presence of 20 nM IGF-1 had significantly more surviving CA3c and CA1 neurons (ANOVA p < 0.001, with post-hoc p < 0.001 in both cases, n = 6 cultures) after DIV 3 than cultures maintained in medium without IGF-1 or other growth factors (Fig. 2a,b).

We measured the amount of lactate dehydrogenase (LDH) released from dead cells into culture medium between DIV 0 and 3. The amount of LDH released by cultures maintained in medium containing IGF-1 was significantly lower than amount of LDH released by cultures in medium without IGF-1 (p < 0.001, n = 6 cultures, Fig. 2c), showing lower neuronal death in the presence of IGF-1. This data suggests that IGF-1 is neuroprotective immediately following brain trauma, in agreement with earlier studies.

### Long-term exposure to IGF-1 was pro-epileptic

**Figure 1:**
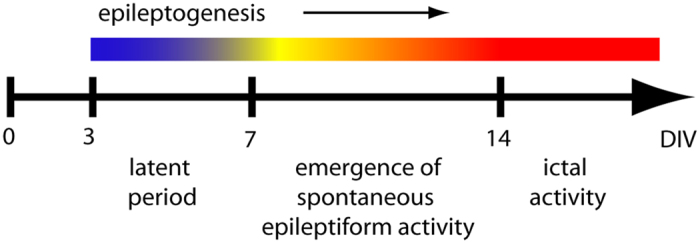
Time course of epileptogenesis in organotypic hippocampal cultures.

**Figure 2:**
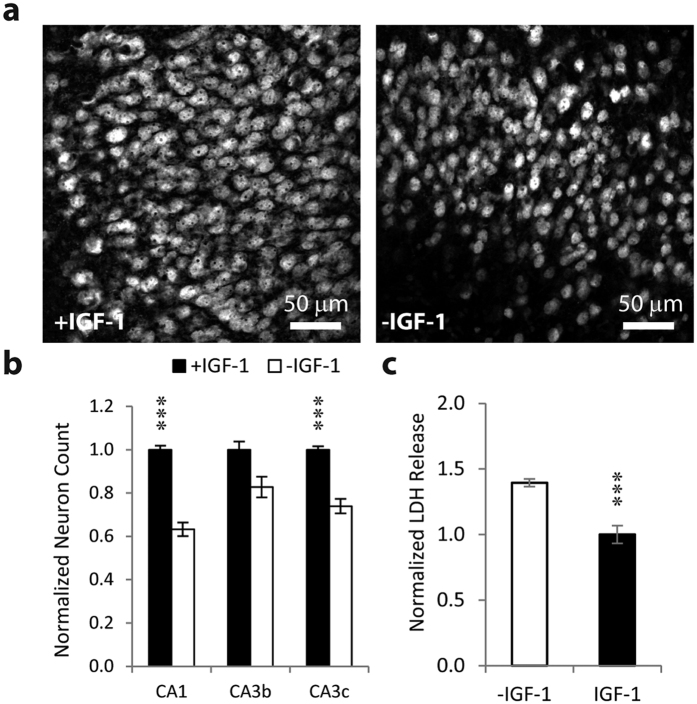
IGF-1 is neuroprotective early after injury. (**a**) Representative micrographs of neurons in area CA3c of organotypic hippocampal cultures at DIV 3 (anti-NeuN stain). Culture in left image has been treated with IGF-1 between DIV 0 and 3, while culture in right image has been treated with vehicle. (**b**) Quantification of surviving neurons in hippocampal areas CA3c, CA3b, and CA1 at DIV 3 in cultures maintained in medium with IGF-1 or vehicle. (**c**) LDH released into culture medium between DIV 0 and 3. Data are represented as mean ± SEM. *represents p < 0.05, ***p < 0.001 versus control (vehicle).

**Figure 3:**
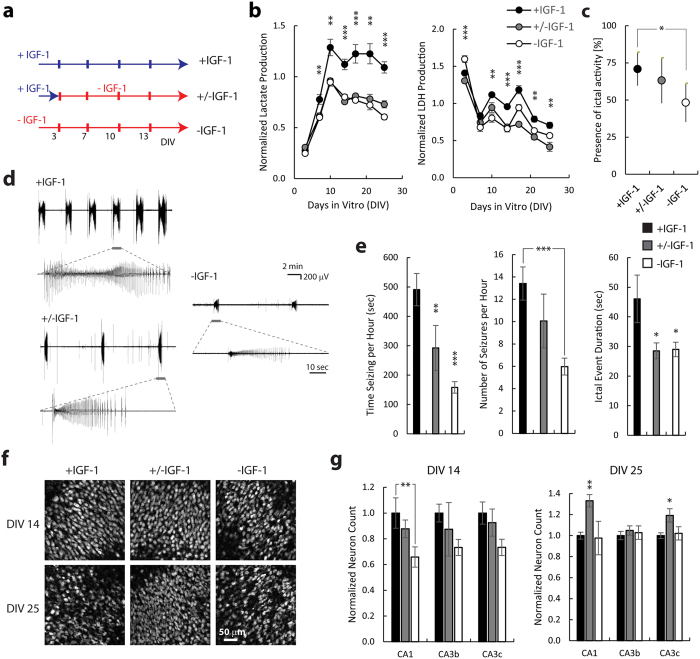
Prolonged, late IGF-1 treatment leads to increased ictal activity and cell death. (**a**) experimental design, (**b**) Lactate (marker of ictal activity) and LDH (marker of cell death) released into the culture medium by IGF-1 treated (+IGF-1) and vehicle-treated (−IGF-1) cultures versus time in culture. (**d**) Representative recordings of electrographic seizures in organotypic cultures on DIV 11–14. Seizure detail is shown in expanded time scale recordings. (**e**) Quantification of ictal event frequency, duration, and time seizing per hour on DIV 11–14. Asterisks indicate significance level of comparisons to +IGF-1 group. (**f**) Representative micrographs of anti-NeuN staining in CA1 layer in cultures on DIV 14 and 25. (**g**) Neuron counts in organotypic cultures on DIV 14 and 25. Asterisks indicate significance of comparison to +IGF-1 group. +IGF-1, +/−IGF-1, and −IGF-1 cultures are represented by black, gray, and white bars, respectively. Data in all panels are represented as mean ± SEM except panel (**c**) where data are presented as a percentage of total and error bars represent 95% confidence interval. *p < 0.05, **p < 0.01, ***p < 0.001.

We then investigated long-term effects of IGF-1 in 3 experimental groups (−IGF-1, +/−IGF-1, and +IGF-1) shown in Fig. 3a. IGF-1 concentration used in all experiments was 20 nM. +/−IGF-1 group was included to control for early IGF-1 neuroprotection. We compared lactate and LDH levels in medium (markers of ictal activity and cell death, respectively^31^) in +IGF-1, +/− IGF-1, and −IGF-1 cultures between DIV 3 and DIV 25 (Fig. 3b). We found that after DIV 7, lactate levels in +IGF-1 cultures were significantly higher than in +/−IGF-1 or −IGF-1 cultures (ANOVA p = 0.009, 0.002, <0.001, <0.001, 0.001, <0.001, and post-hoc p = 0.024/0.015, 0.005/0.004, <0.001/<0.001, 0.001/<0.001, 0.004/0.002, <0.001/<0.001 for +IGF-1 vs. +/−IGF-1/−IGF-1 on DIV 7, 10, 14, 17, 21, and 25, respectively, n = 4), suggesting that ictal activity was increased by prolonged IGF-1 treatment. LDH levels were higher on DIV 3 in −IGF-1 cultures compared to +IGF-1 and +/− IGF-1 cultures (ANOVA p < 0.001, post-hoc p = 0.008 for +IGF-1 vs. −IGF-1 comparison, and p < 0.001 for +/−IGF-1 vs. −IGF-1 comparison, n = 4), confirming results shown in Fig. 2. Significantly higher LDH levels were measured in medium from +IGF-1 cultures after DIV 10 (ANOVA p = 0.007, <0.001, <0.001, 0.001, 0.005, and post-hoc p = 0.134/0.006, <0.001/<0.001, <0.001/0.011, 0.001/0.017, 0.004/0.173 for +IGF-1 vs. +/−IGF-1/−IGF-1 on DIV 10, 14, 17, 21, 25 respectively, n = 4). We confirmed the lactate data with extracellular recording of spontaneous activity in cultures between DIV 11 and DIV 14 (Fig. 3c–e). This period corresponds to the emergence of spontaneous ictal activity in this model of epilepsy^31^,^36^ (Fig. 1), and a period marked by increased lactate production (Fig. 3b). We recorded 62 +IGF-1, 38 +/− IGF-1, and 56 −IGF-1 cultures, and found that fewer −IGF-1 cultures had ictal activity during the 45 minute analysis period than +IGF-1 cultures (all three groups χ^2^ = 6.505, p = 0.039, z-test p = 0.02 for +IGF-1 vs. –IGF-1 comparison, Fig. 3c). On the other hand, there was no difference between the percentages of +IGF-1 and +/−IGF-1 cultures that had ictal activity (z-test p = 0.554). However, when analyzing seizing cultures only, we found that +IGF-1 cultures spent more time seizing than either +/−IGF-1 or −IGF-1 cultures (ANOVA p = 4.4357*10^−5^, p = 0.004 for +IGF-1 vs. +/−IGF-1 comparison, p < 0.001 for +IGF-1 vs. −IGF-1 comparison, n = 44 +IGF-1, 24 +/−IGF-1, and 27 −IGF-1 cultures with ictal activity). We also found that +IGF-1 cultures had more electrographic seizures per hour than −IGF-1 cultures (ANOVA p = 0.005, post-hoc p < 0.001), while both +/−IGF-1 and −IGF-1 cultures had shorter ictal events than +IGF-1 cultures (ANOVA p = 0.022, post-hoc p = 0.033 for +IGF-1 vs. +/−IGF-1 comparison, and p = 0.016 for +IGF-1 vs. −IGF-1 comparison). No significant differences were found when comparing ictal activity in +/−IGF-1 and −IGF-1 cultures (post-hoc p = 0.932, 0.865, 0.925 for comparisons of time seizing per hour, number of seizures per hour, and ictal event duration). We then compared numbers of surviving neurons in CA1, CA3b, and CA3c regions of organotypic cultures on DIV 14 and DIV 25 (Fig. 3f,g). On DIV 14, there were more neurons in CA1 of +IGF-1 cultures compared to −IGF-1 cultures (ANOVA p = 0.022, post-hoc p = 0.009, n = 7, 3, 8 +IGF-1, +/−IGF-1, and −IGF-1 cultures, respectively). On DIV 25, there were more neurons in CA1 and CA3c of +/−IGF-1 cultures than +IGF-1 (ANOVA p < 0.001, 0.712, 0.024 for CA1, CA3b, and CA3c, respectively, post-hoc p = 0.002 and 0.029 for CA1 and CA3c, n = 10, 10, 4). There were also more neurons in CA1 of +/−IGF-1 cultures compared to −IGF-1 cultures (p = 0.011).

### Short-term exposure to IGF-1 had no effect on seizures

**Figure 4:**
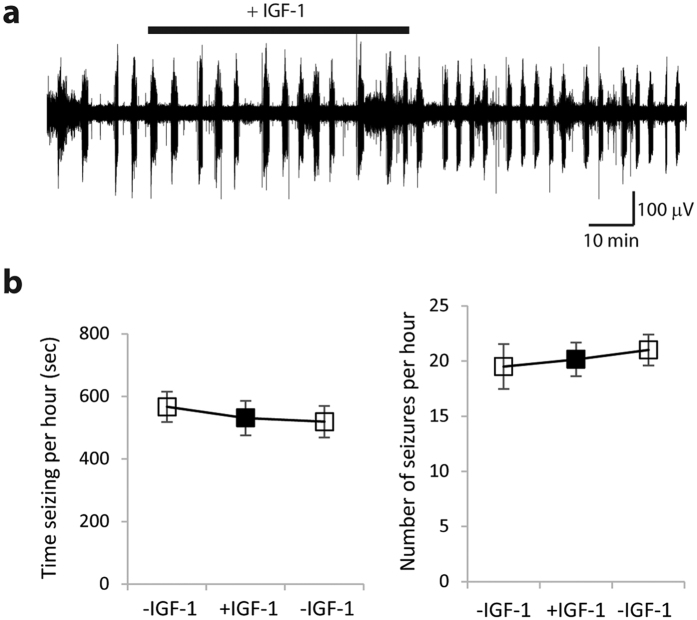
Short-term application of IGF-1 has no effect on ictal activity. (**a**) Representative recording of electrographic ictal events in organotypic hippocampal culture at DIV 11–14. Time of +IGF-1 application is indicated by the black bar. (**b**) Quantification of time seizing per hour (left) and number of seizures per hour (right) prior, during, and after +IGF-1 application. Data are represented as mean ± SEM.

We examined the influence of short-term application of IGF-1 on ictal activity, to determine if pro-epileptic activity of IGF-1 was due to an acute pro-convulsant effect. Organotypic hippocampal cultures maintained with no IGF-1 from DIV 3 to DIV 11–14 (same condition as +/−IGF-1 cultures in Fig. 3a), were used for this experiment. Cultures were placed into artificial cerebrospinal fluid (ACSF), and ictal activity was measured before and during IGF-1 wash-in, and after IGF-1 wash-out (Fig. 4). We determined that activation (phosphorylation) of IGF-1R in organotypic hippocampal cultures occurs within 30 minutes of addition of 20 nM IGF-1 to culture medium (Supplementary Fig. 1). The duration of 20 nM IGF-1 wash-in was set to 1 hour, to ensure that IGF-1 exposure was long enough for IGF-1R signaling cascade activation. The amount of time seizing per hour was not significantly different before, during, or after acute IGF-1 application (paired t-test, −IGF-1 to +IGF-1, p = 0.582; +IGF-1 to −IGF-1, p = 0.368, n = 14 cultures). Number of seizures per hour was also not significantly different before, during, or after acute IGF-1 application (paired t-test, −IGF-1 to +IGF-1 p = 0.806; +IGF-1 to −IGF-1 p = 0.541, n = 14 cultures).

### IGF-1R, Akt, MAPK, and S6 signaling

**Figure 5:**
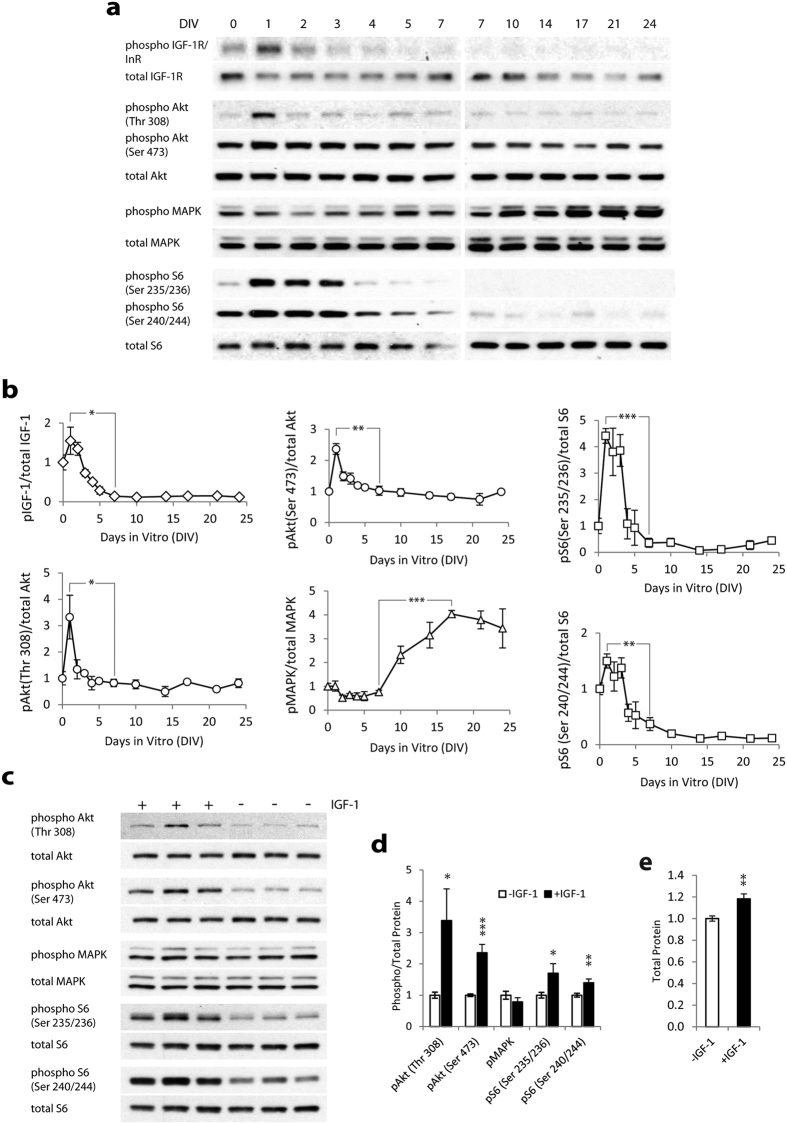
IGF-1, Akt, MAPK, and S6 signaling. (**a**) Representative Western Blots of protein phosphorylation in −IGF-1 cultures between 0 and 24 DIV. (**b**) Quantification of the time course of protein phosphorylation in −IGF-1 cultures. (**c**) Representative Western Blots of phosphorylated and total Akt, MAPK, and S6 on DIV 6. Cultures treated with IGF-1 are indicated with (+), cultures treated with vehicle are indicated with (−). (**d**) Quantification of corresponding band densities. Ratios for vehicle-treated cultures were used for normalization. (**e**) Comparison of total (all) protein detected in single culture lysates revealed that IGF-1 treated cultures have higher protein content than vehicle treated cultures. Data are represented as mean ± SEM. *p < 0.05, **p < 0.01, ***p < 0.001.

We determined the time course of IGF-1R, Akt, MAPK, and S6 phosphorylation in −IGF-1 cultures (Fig. 5a,b). We measured phosphorylation of IGF-1R at Tyr 1135/1136 (anti-phospho IGF-1R antibody (Supplementary Fig. 1) that also detects phosphorylation of the insulin receptor(InR)), Akt at Thr 308 and Ser 473, MAPK at Thr202/Tyr204, and S6 at Ser 235/236 and Ser 240/244 (n = 3 cultures, all time points). We found that there was a trend toward increased phosphorylation of IGF-1R/InR (not statistically significant, p = 0.239), Akt at Thr 308 (p = 0.055), and significant increase in phosphorylation of Akt at Ser 473 (p = 0.002) and S6 at Ser 235/236 (p = 0.001) and Ser 240/244 (p = 0.039) between DIV 0 (slices before culturing) and DIV 1. Phosphorylation of these proteins significantly decreased by DIV 7 (p = 0.016, 0.041, 0.004, <0.001, 0.003 for DIV 1 to 7 comparison of phospho IGF-1R/InR, phospho Akt (Thr 308 and Ser 473), phospho S6 (Ser 235/236 and Ser 240/244), respectively). On the other hand, there were no significant changes in phosphorylation of MAPK from DIV 0 to 1 (p = 0.955) or DIV 1 to 7 (p = 0.318). But, MAPK phosphorylation underwent a significant late increase (p < 0.001, DIV 7 vs. 17).

### Long-term IGF-1 treatment activated Akt and S6, but not MAPK signaling, and increased protein synthesis

We determined downstream signaling activated by prolonged exposure to IGF-1. All cultures received IGF-1 from DIV 0 to DIV 3, and were then maintained in the presence or absence of IGF-1 from DIV 3 to DIV 6. This time period was chosen so that effects of ictal activity and seizure-dependent cell death that spontaneously occur in this model after DIV 7 were minimized. We found that prolonged (3 days) exposure to IGF-1 caused an 18 ± 5% increase in the amount of protein contained in organotypic hippocampal cultures suggesting increased protein synthesis (Fig. 5e, p = 0.002, n = 12). We also found that prolonged exposure to IGF-1 caused significant increase in Akt phosphorylation at Thr 308 (p = 0.029), and at Ser 473 (p < 0.001), and in S6 phosphorylation at Ser 235/236 (p = 0.036) and Ser 240/244 (p = 0.009) (Fig. 5c,d). MAPK phosphorylation was not significantly affected by prolonged IGF-1 (p = 0.256). We used n = 12 cultures from 4 animals for this experiment.

### Increase in ictal activity due to IGF-1 was prevented by inhibition of Akt or mTOR

**Figure 6:**
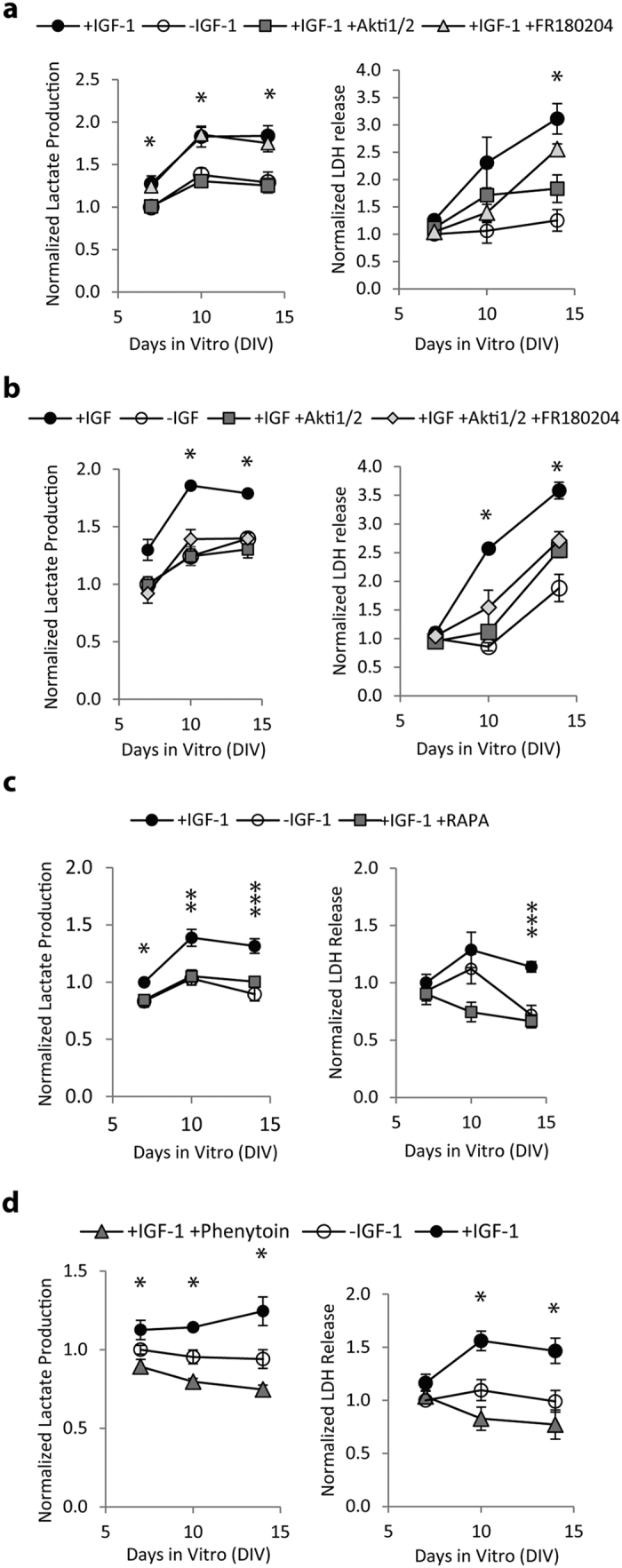
Effect of Akt, MAPK, and mTOR inhibitors, and anticonvulsant phenytoin, applied starting from DIV 3, on epileptogenesis. (**a**) Lactate (marker of ictal activity) and LDH (marker of cell death) released into culture medium by Akti1/2 (Akt inhibitor) and FR180204 (MAPK inhibitor) +IGF-1 cultures. A control (no IGF-1 since DIV 3, no inhibitor) group is included for comparison. (**b**) Combined Akt and MAPK inhibitors do not have a different effect than Akt inhibitor alone. (**c**) Rapamycin (RAPA) was applied to +IGF-1 cultures starting from DIV 3, and significantly reduced lactate and LDH. (**d**), Phenytoin added to IGF-1 treated cultures significantly decreases both lactate and LDH. −IGF-1 groups in (**a–d**) had IGF-1 applied between DIV 0 and 3, and were then maintained in the absence of IGF-1 (equivalent to +/−IGF-1 group in Fig. 3a). Data are represented as mean ± SEM. *p < 0.05, **p < 0.01, ***p < 0.001.

Akt 1/2 kinase inhibitor (Akti ½, 1 μM), inhibitor of Akt, and FR 180204 (10 μM), inhibitor of MAPK (ERK1/2), were used to examine the role of Akt and MAPK signaling in IGF-1 induced increase in ictal activity. We verified that inhibitors reduced phosphorylation of their target protein at the concentrations used (Supplementary Fig. 2). Inhibitors were added starting from DIV 3 to cultures maintained in the presence of IGF-1, and levels of lactate and LDH in medium were compared to +IGF-1 and +/−IGF-1 cultures between DIV 6 and 14 (Fig. 6a). We found that Akt inhibitor significantly reduced lactate levels on DIV 6, 10, and 14 (ANOVA p < 0.001 for all days, post-hocp = 0.007, 0.011, 0.012 respectively), and LDH level on DIV 14 (ANOVA p < 0.001, post-hoc p = 0.003), while MAPK inhibitor (FR 180204) did not have significant effects compared to +IGF-1 cultures. We used n = 6 cultures per condition.

While MAPK inhibition was not statistically significant, we noticed that LDH levels were somewhat lower in FR 180204 treated +IGF-1 cultures, compared to vehicle treated +IGF-1 cultures, on DIV 10 and 14 (Fig. 6a, right panel). We hypothesized that inhibition of Akt and MAPK signaling may have synergistic neuroprotective effect in IGF-1 treated cultures. Therefore, we compared lactate and LDH levels between DIV 6 and DIV 14 in cultures treated with +IGF-1, +/−IGF-1, +IGF-1 with Akt inhibitor, and +IGF-1 with Akt and MAPK inhibitors (Fig. 6b). This experiment confirmed the effect of Akt inhibitor on lactate and LDH levels compared to +IGF-1 cultures, but no significant differences were observed between +IGF-1 with Akti 1/2 and +IGF-1 with Akti 1/2 and FR180204 groups (n = 3 cultures per condition).

We then investigated if rapamycin, an inhibitor of mTOR (downstream of Akt), affects IGF-1 contribution to epileptogenesis. We found that 20 nM of rapamycin significantly reduces lactate production between DIV 6 and DIV 14 in cultures treated with +IGF-1 to levels found in +/−IGF-1 cultures (ANOVA p = 0.015, 0.002, <0.001 on DIV 6, 10 and 14, respectively, with post-hoc p = 0.014, 0.002, and 0.001, n = 6 cultures per condition). We also found that rapamycin significantly reduced cell death, as measured by LDH release, on DIV 14 in +IGF-1 cultures (ANOVA p < 0.001, post-hoc p < 0.001, n = 6 cultures per condition).

### Anticonvulsant phenytoin suppressed IGF-1 induced increases in ictal activity and cell death

Phenytoin (100 μM) was added to +IGF-1 cultures starting from DIV 3 and lactate and LDH levels in culture medium between DIV 7 and 14 were compared to +IGF-1 and +/−IGF-1 cultures (Fig. 6d). We found that phenytoin significantly reduced lactate levels (ANOVA p = 0.011 for DIV 7, and p < 0.001 for DIV 10 and 14, post-hoc Bonferroni p = 0.01, <0.001, <0.001 between +IGF-1 and +IGF-1 + phenytoin groups on DIV 7, 10, and 14, respectively), and LDH levels on DIV 10 and 14 (ANOVA p < 0.001, p = 0.003, and post-hoc Bonferroni p < 0.001, p = 0.003, respectively, when comparing +IGF-1 and +IGF-1 + phenytoin groups). We used n = 6 cultures per condition.

## Discussion

Our principal findings were that IGF-1 is neuroprotective immediately after injury, but prolonged treatment with IGF-1 leads to an increase in electrographic seizures. Short-term IGF-1 treatment had no effect on ictal events. Prolonged IGF-1 treatment led to increased phosphorylation of Akt and S6, and increased protein synthesis, while inhibition of Akt or mTOR prevented an increase in ictal activity caused by IGF-1.

In organotypic hippocampal cultures, the initial period after injury, DIV 0–3, is characterized by a high amount of cell death that decreases by DIV 4–7^36^,^37^, mirroring the time course in animal models of traumatic brain injury^38^,^39^,^40^. Our finding that IGF-1 is neuroprotective during this early period is in agreement with results obtained from short-term IGF-1 treatments in animal models of brain injury^10^,^11^,^41^. Interestingly, increased phosphorylation of IGF-1R/InR, Akt, and S6 in −IGF-1 organotypic cultures after first 24 hours *in vitro* (Fig. 5a,b) closely mirrors increased phosphorylation of these proteins in animal models of traumatic brain injury^10^,^19^. This finding suggests the presence of injury-activated autocrine or paracrine growth factor signaling in the hippocampal slice cultures up to 72 hours after slicing.

The initial period is followed by a latent period when there is little cell death, and neural activity includes single and multiple units and population spikes. The population spikes become more frequent by DIV 6, when spontaneous ictal events begin to occur^36^ (Fig. 1). To examine the role of IGF-1 in epileptogenesis, we used three experimental groups (Fig. 3a). We included +/−IGF-1 group to control for differences in the number of surviving neurons in +IGF-1 and −IGF-1 groups by DIV 3. We found that cultures in all 3 groups developed epilepsy, demonstrating that externally applied IGF-1 is not necessary for development of spontaneous population spikes or ictal events. The fraction of –IGF-1 cultures with ictal events was smaller than corresponding fraction of +IGF-1 cultures, but −IGF-1 cultures also had fewer surviving neurons (Figs 2 and 3f,g), and this may have affected epileptogenesis. However, we discovered that prolonged treatment with IGF-1 increased ictal activity not only when compared to −IGF-1 cultures, but also when compared to +/−IGF-1 cultures that entered this period with same number of neurons as +IGF-1 cultures (Fig. 3f,g). It was recently reported that acute application of IGF-1 enhances chemically induced seizures^42^, in contrast to our finding that acute application of IGF-1 has no effect (Fig. 4). This difference is likely due to different mechanisms of IGF-1 effect on seizures induced by chemoconvulsants versus spontaneously occurring seizures in our model.

Ictal activity in organotypic cultures leads to a second wave of cell death that peaks around DIV 14–17, and that is significantly attenuated by addition of glutamate antagonist kynurenic acid or anticonvulsant phenytoin to culture medium^36^,^37^. Interestingly, phosphorylation of MAPK in −IGF-1 cultures increased during this period, suggesting that MAPK signaling may be activated by ictal cell death (Fig. 5a,b). We found that IGF-1 increased cell death during this time *in vitro*, in direct contrast to neuroprotective role of early IGF-1 treatment. This cell death was likely caused by increased ictal activity rather than directly by IGF-1, since addition of anticonvulsant phenytoin to IGF-1 treated cultures eliminated the increase in toxicity (Fig. 6d).

We examined the potential downstream effectors of IGF-1 signaling in this model by looking at phosphorylation of Akt, MAPK, and S6 at the conclusion of the latent period (DIV 6). At this time, epileptogenic processes are actively ongoing^43^, but ictal activity and concomitant cell death have not yet occurred. Thus, we can separate IGF-1 effects from potential changes in intracellular signaling that may be introduced by epileptiform activity or cell death at later time points. We found that IGF-1 treatment during the latent period increases phosphorylation of Akt and S6, but not MAPK. It was previously found that promotion of neuronal survival by IGF-1 is mediated by Akt^44^. In contrast, our results suggest that IGF-1 - Akt signaling can lead to neuronal death through increased ictal activity. and that effects of IGF-1 signaling are context-dependent as has been previously found in other models^13^,^45^.

Akt is an upstream activator of mTOR, while S6, a ribosomal protein that is involved in translation, is activated by mTOR through S6 kinase^20^,^46^,^47^. We previously found that mTOR inhibition in organotypic hippocampal cultures is antiepileptic^31^ and we also found that mTOR inhibition is sufficient to prevent an increase in ictal activity and subsequent neuron death caused by IGF-1 in this work. It should be noted that some S6 phosphorylation was present without any externally applied IGF-1 (Fig. 5c), suggesting that other pathways may also play a role in activation of mTOR signaling in this model. However, increased activation of Akt and S6 due to IGF-1 treatment, and increased protein synthesis (hallmark of mTOR activation^48^), suggest that the mTOR pathway may mediate pro-epileptic effects of IGF-1.

Prolonged IGF-1 application resulted in increased amount of protein by DIV 6, when both +IGF-1 and −IGF-1 (with no IGF-1 since DIV 3) cultures have had similar levels of cell death (Fig. 3b), suggesting that the increased protein in tissue lysates on DIV 6 is due to increased protein synthesis, and not to improved neuronal survival. It has been previously suggested that IGF-1 receptor is involved in regulation of protein synthesis and neural metabolism^3^. IGF-1 plays a major role in central nervous system development, from proliferation, differentiation, brain size^49^ and establishment of neuronal polarity^50^ to postnatal neurogenesis and synaptogenesis^51^,^52^, and axon outgrowth^53^,^54^. It has been proposed that the high IGF-1 mRNA expression in neurons that undergo dendritic and synaptic remodeling plays a role in trophic support of neurite outgrowth and synaptogenesis^8^. Axonal sprouting, dendritic plasticity, and neurogenesis have been identified as structural changes that play a role in epileptogenesis after brain insult^55^. Inhibition of mTOR in models of acquired epilepsy led to reduction in mossy fiber sprouting, as well as a decrease in seizures in some but not all preparations^31^,^46^,^47^,^56^,^57^,^58^,^59^. Addition of external IGF-1 to organotypic hippocampal culture, which is injured brain tissue that is undergoing axon sprouting, dendritogenesis and synaptogenesis^31^,^60^,^61^,^62^ may alter these processes through activation of anabolic mTOR signaling^63^, potentially exacerbating epileptogenesis. In addition, we must consider that neuroprotection may sometimes save neurons that would be “better off dead”. That is to say, it may be better not to rescue some populations of neurons, such as those that have been severely injured or that are receiving abnormal inputs as a consequence of erroneous synaptogenesis. This is only a speculation at this point, but this possibility emphasizes the need to understand in more detail what populations of neurons are salvaged by different neuroprotective strategies. For example, it may be that IGF-1 should only be administered during a specific time window after injury.

On the other hand, both recovery and epileptogenesis may be enhanced by nonspecific anabolic enhancement after injury. Axon remodeling, neurogenesis, and synaptogenesis have been identified as potential targets for pharmacological interventions to promote brain repair and regeneration after traumatic brain injury^64^. Long-term (4 to 21 days) addition of IGF-1 was found to be beneficial in alleviating neurobehavioral deficits after traumatic brain injury^12^,^65^ and ischemic injury^66^. To reconcile the beneficial and pro-epileptic effects of IGF-1, varying incidence rates and time-courses of epileptogenesis after different types of brain injury need to be considered. Stroke increases epilepsy risk 20-fold, while increase in risk due to traumatic brain injury ranges from less than 2-fold increase in cases of mild civilian head injury to over 500-fold for military penetrating head injury^32^. Animal models of brain injury also have variable incidence of epilepsy and latency to first occurrence of spontaneous seizures that span the range from 6 to 50% of animals that develop epilepsy after 4 weeks to 6 months^67^. On the other hand, epileptogenesis occurs in most of organotypic cultures after 1 to 2 weeks^36^. Therefore, in this model, pro-epileptic effects of long-term IGF-1 treatment are apparent after 2 weeks, while same effects in animal models may be detectable only after several months. Also, we found that while IGF-1 is not necessary for epileptogenesis, it increases severity of epileptic events in tissue that is already epileptic. It may be possible that prolonged IGF-1 treatment does not increase incidence of epilepsy, and animals that will not develop spontaneous seizures may still derive neurobehavioral benefit from increased IGF-1. If this is confirmed by future studies, clinical implication will be that long-term treatment with IGF-1 or its agonists may be beneficial in cases where brain injury carries relatively low risk of epilepsy, but harmful in cases where epilepsy risk is high.

In conclusion, this study provides evidence that IGF-1 may play a role in epileptogenesis. Effects of IGF-1 application were strongly context-dependent, with IGF-1 increasing neuronal survival immediately after injury, but contributing to epileptogenesis after prolonged application. Chronic exposure to IGF-1 led to an increase in ictal event-mediated cell death, in contrast to early IGF-1 neuroprotection. Pro-epileptogenic effects of IGF-1 were mediated by Akt-mTOR, but not MAPK signaling.

## Methods

### Organotypic cultures

Hippocampal slices with a thickness of 350 μm were dissected from the hippocampus of 7 day old Sprague-Dawley rats. After dissection, all slices were maintained on poly-D-lysine coated six-well tissue culture plates on a rocking platform in a 37 °C incubator with humidified atmosphere containing 5% CO_2_. Culture medium consisted of Neurobasal-A supplemented with 0.5 mM GlutaMAX, 30 μg/ml gentamicin (all from Life Technologies), 250 μg/ml bovine serum albumin and 14 ng/ml sodium selenite (Sigma). IGF-1 and inhibitors were added to the medium at time points and in concentrations specified in the appropriate sections. Medium was changed twice a week. Cultures were discarded if damage to a portion of pyramidal layer or granule cell layer was visible using bright-field microscopy. All animal use protocols were approved by the Institution Animal Care and Use Committee (IACUC) at Lehigh University and were conducted in accordance with the United States Public Health Service Policy on Humane Care and Use of Laboratory Animals.

### Lactate and lactate dehydrogenase (LDH) assays

Culture supernatant was collected during medium changes. Lactate and LDH relative concentrations in the supernatant were determined by using kits (Eton Bioscience and Roche Diagnostics, respectively) according to manufacturers’ protocols.

### Electrical recordings

Local field potentials were recorded with a tungsten microelectrode placed into area CA1 of organotypic hippocampal cultures. Interface chamber was used to record spontaneous epileptiform activity in original culture medium (warmed up 37 °C and balanced in 5% CO_2_) for comparison of IGF-1 and vehicle-treated cultures. For IGF-1 wash-in and wash-out experiments, cultures were transferred to a conventional submerged chamber and continuously superfused with oxygenated artificial cerebrospinal fluid (ACSF, 95% O_2_ and 5% CO_2_) at 37 °C and a flow rate of 1 ml/min. ACSF contained 126 mM NaCl, 25 mM NaHCO_3_, 3.5 mM KCl, 1.3 mM MgCl_2_, 2 mM CaCl_2_, and 11 mM D-glucose. Recorded signals were amplified by 1000x (band-pass 1 Hz to 5 kHz) and digitized using multichannel analog-to-digital signal acquisition board and LabVIEW software (National Instruments).

### Western Blots

Organotypic cultures were scraped from tissue culture plates and homogenized in RIPA lysis buffer that contained protease and phosphatase inhibitors (Thermo Scientific). Protein content of culture lysates was measured using Micro BCA protein assay kit (Thermo Scientific). Proteins were separated via electrophoresis in 12% Tris-Glycine Mini Gels (Life Technologies), transferred to a PVDF membrane, and stained with antibodies (1:10,000 rabbit antibodies to phospho-Akt (Ser473) (D9E), phospho-Akt (Thr308) (C31E5E), phospho-p44/42 MAPK (ERK1/2) (Thr202/Tyr204) (D13.14.4E), phospho-S6 (Ser235/236) (D57.2.2E), phospho-S6 (Ser 240/244) (D68F8), phospho−IGF-1Rβ (Tyr1135/1136)/Insulin Receptor β (Tyr1150/1151) (19H7, used at 1:500 concentration), total Akt (C67E7), p44/42 MAPK (Erk1/2) (137F5), S6 Ribosomal Protein (5G10), and IGF-1Rβ (D23H3, used at 1:1000 concentration), all from Cell Signaling Technology, and anti-rabbit horseradish peroxidase-conjugated secondary antibody from Jackson ImmunoResearch Laboratories). Bands were visualized using Pierce enhanced chemiluminescence (ECL) substrate on CL-XPosure X-ray films (Thermo Scientific), scanned, and quantified using densitometry via ImageJ software (NIH).

### Immunostaining

Organotypic cultures were fixed, scraped from tissue culture plates, washed, and permeabilized. Anti-NeuN antibody conjugated to a fluorophore (Millipore) was applied to cultures, which were then washed, mounted, and imaged with a confocal microscope (Zeiss LSM 510 META). Optical stacks spanning the entire thickness of the culture were acquired. Cells were counted using cell counter plugin in ImageJ. Cell counts were normalized by the length of the pyramidal layer of the cultured slice (Supplementary Fig. 3).

### Statistics and electrographic analysis

Ictal and interictal events were defined as described earlier^31^. Quantification was performed on digitized data in Matlab using a sliding 10 second window to identify start and end of individual ictal events. Minimum sample sizes were calculated for continuous variable and repeat studies^68^ and used in corresponding experiments. Data were tested for normality using Kolmogorov-Smirnov test in SigmaPlot. Two-tailed Student’s t-test was used for two-variable comparisons, paired t-test was used for before-after comparisons of IGF-1 wash-in and wash-out, and one-way ANOVA with Bonferroni post hoc analysis was used for multiple variable comparisons. Data that did not pass normality test were analyzed with Mann-Whitney Rank Sum test or ANOVA on Ranks. Quantitative data were expressed as mean ± SEM in text and figures except as indicated. Statistical significance was defined as p < 0.05.

## Additional Information

**How to cite this article**: Song, Y. *et al*. Neuroprotective levels of IGF-1 exacerbate epileptogenesis after brain injury. *Sci. Rep.* **6**, 32095; doi: 10.1038/srep32095 (2016).

## Supplementary Material

Supplementary Information
