# Supplementary material for: Neuroprotective levels of IGF-1 exacerbate epileptogenesis after brain injury
Source: Sci Rep. 2016 Aug 26;6:32095. doi: 10.1038/srep32095 (PMC4999804; doi:10.1038/srep32095)
Supplement: Supplementary Information [file srep32095-s1.pdf]

Supplementary Information for manuscript "Neuroprotective levels of IGF-1 exacerbate epileptogenesis after brain injury" by Y. Song, C. Pimentel, K. Walters, L. Boller, S. Ghiasvand, J. Liu, K.J. Staley, and Y. Berdichevsky.

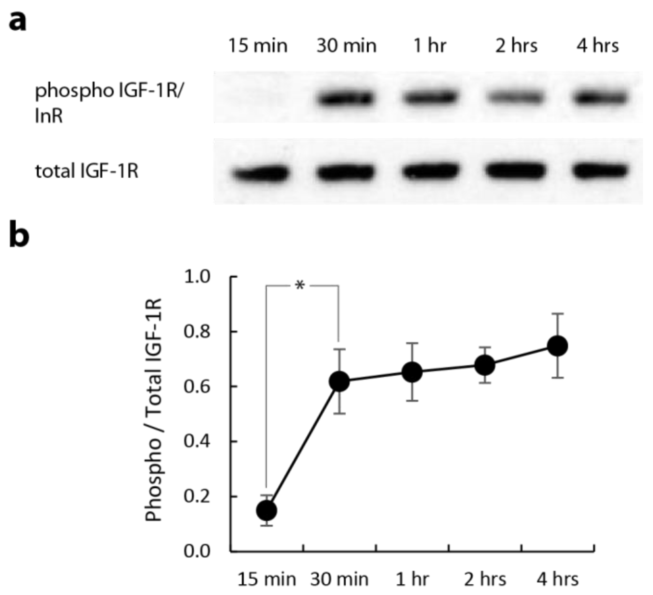

Supplementary Figure 1. Time course of IGF-1R phosphorylation after addition of IGF-1 to the culture medium of -IGF-1 cultures on DIV 3. Antibody that detects phosphorylated IGF-1R as well as InR was used. No insulin was present in the culture medium. IGF-1R is significantly more phosphorylated after 30 minutes of IGF-1 application ($p = 0.011$, $n = 4$). Increase of phospho IGF-1R / total IGF-1R ratio from 30 minutes to 4 hours was not significant ($p = 0.463$, $n = 4$). Data represented as mean ± SEM, *$p < 0.05$.

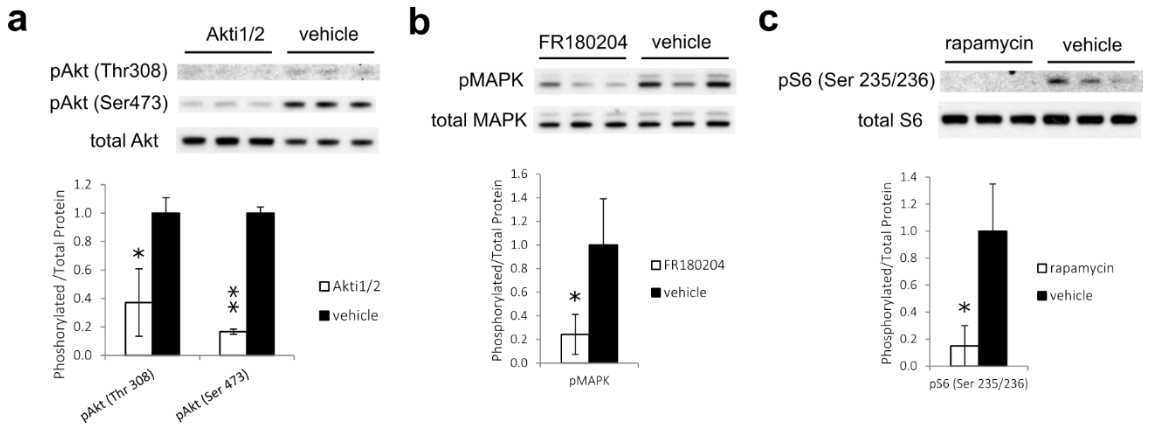

Supplementary Figure 2. Akti1/2, FR180204, and rapamycin inhibit phosphorylation of their target proteins. Inhibitors were applied on DIV 3 to +IGF-1 cultures, and lysates were collected on DIV 6. 1 µM Akti1/2 significantly reduced phosphorylation of Akt at Thr 308 ($p = 0.014$) and at Ser 473 ($p < 0.001$). 10 µM of FR180204 significantly reduced phosphorylation of MAPK ($p = 0.037$), and 20 nM rapamycin significantly reduced phosphorylation of S6 ($p = 0.018$). n = 3 cultures, all inhibitors and conditions. Data represented at mean ± standard deviation.

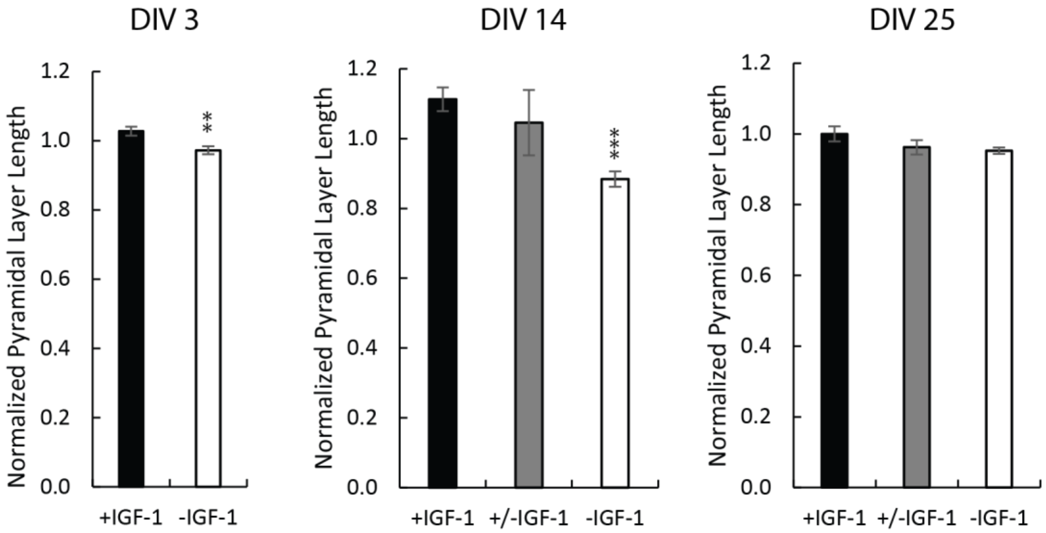

Supplementary Figure 3. Comparison of pyramidal layer length (CA1+CA3b+CA3c) in organotypic hippocampal cultures with different IGF-1 treatments. On DIV 3, vehicle-treated cultures (-IGF-1) had a significantly shorter pyramidal layer ($p = 0.009$, $n = 6$). On DIV 14, pyramidal layer in –IGF-1 cultures was also significantly shorter than in +IGF-1 cultures (ANOVA $p = 0.005$, post-hoc $p < 0.001$ for +IGF-1 vs. – IGF-1 comparison, n = 7, 3 and 8 for +IGF-1, +/-IGF-1, and -IGF-1 cultures, respectively). No significant differences were found on DIV 25 (ANOVA $p = 0.292$, n = 10, 10, and 4 for +IGF-1, +/-IGF-1, and -IGF-1 cultures, respectively). Data represented as mean ± SEM, ** $p < 0.01$, *** $p < 0.001$.
